# HiC-GNN: A generalizable model for 3D chromosome reconstruction using graph convolutional neural networks

**DOI:** 10.1016/j.csbj.2022.12.051

**Published:** 2022-12-31

**Authors:** Van Hovenga, Jugal Kalita, Oluwatosin Oluwadare

**Affiliations:** a Department of Mathematics, University of Colorado, Colorado Springs, CO, United States; b Department of Computer Science, University of Colorado, Colorado Springs, CO, United States

**Keywords:** Hi-C, 3D chromosome structure, Graph neural networks, Chromosome conformation capture, 3D genome

## Abstract

Chromosome conformation capture (3 C) is a method of measuring chromosome topology in terms of loci interaction. The Hi-C method is a derivative of 3 C that allows for genome-wide quantification of chromosome interaction. From such interaction data, it is possible to infer the three-dimensional (3D) structure of the underlying chromosome. In this paper, we developed a novel method, HiC-GNN, for predicting the 3D structures of chromosomes from Hi-C data. HiC-GNN is unique from other methods for chromosome structure prediction in that the models learned by HiC-GNN can be generalized to data that is distinct from the training data. This aspect of HiC-GNN allows models that were trained on one Hi-C contact map to be used for inference on entirely different maps. To the authors’ knowledge, this generalizing capability is not present in any existing methods. HiC-GNN uses a node embedding algorithm and a graph neural network to predict the 3D coordinates of each genomic loci from the corresponding Hi-C contact data. Unlike other methods, our algorithm allows for the storage of pre-trained parameters, thus enabling prediction on data that is entirely different from the training data. We show that our method can accurately generalize a single model across Hi-C resolutions, multiple restriction enzymes, and multiple cell populations while maintaining reconstruction accuracy across three Hi-C datasets. Our algorithm outperforms the state-of-the-art methods in accuracy of prediction and runtime and introduces a novel method for 3D structure prediction from Hi-C data. All our source codes and data are available at https://github.com/OluwadareLab/HiC-GNN.

## 1 Introduction

The structure of chromosomes is known to influence several genomic functions [1], [2], [3]. Thus, discovering the three-dimensional (3D) structure of chromosomes is important for understanding the functional and regulatory elements of genomes. For this reason, chromosome conformation capturing techniques such as 3 C [4], 4 C [5], 5 C [6], and Hi-C [7], [8], [9] were developed to analyze the spatial organization of chromatins in a cell. In general, chromosome conformation capture relies on quantification of contacts between genomic loci to give insight into the structural organization of the genome. Hi-C is a chromosome conformation capture technology that allows for all-to-all quantification of intra-genomic contacts, i.e., contacts are measured between each pair of loci within the genome. This is accomplished via the following steps [7], [8], [9]. First, chromatin between several chromosomes are cross linked using a fixative solution. Then, the chromatin is isolated and digested by an enzyme. This results in pairs of crosslinked DNA fragments that may differ linearly but are close in physical space. These separate fragments are then re-ligated, and the crosslinks are reversed, thus resulting in templates. These templates are then amplified and interrogated, usually using polymerase chain reaction (PCR) and DNA sequencing. The resulting data describes the frequency of ligation junctions between genomic loci. These relative contact frequencies describe the proximity of the loci in 3D space. Due to its all-to-all nature, the Hi-C method allows for global insight into the spatial organization of entire genomes.

The high quantity of data that is produced with the Hi-C method has led to the development of several computational methods that aim to make inference of the 3D structure of chromosomes from their respective Hi-C data [10]. A strategy often employed by these computational methods is the distance-restraint optimization strategy [11], [12], [13], [14], [15], [16]. Usually, the distance-restraint method converts the contacts of the input Hi-C map to distances using an inverse power law [9]. These distances are typically referred to as wish distances. Following this conversion step, a set of xyz coordinates is initialized; each xyz coordinate corresponds to a locus in the chromosome. The model is then trained by optimizing these xyz coordinates so that the pairwise Euclidean distances of the predicted structure accurately recreate the wish distances of the input.

### 1.1 Motivation

There are several limitations associated with traditional distance-restraint methods. Firstly, some distance-restraint methods assume that chromosomal contacts are independent and identically distributed [11] This assumption is false since self-attracting nature of polymers results in correlations between neighboring contact sites [17]. Moreover, ignoring intra-contact correlations removes a potentially valuable source of information for structure prediction. The second limitation associated with distance-restraint methods is that, to the authors' knowledge, all current distance-restraint methods are instance-based. That is, to predict the structure of a fixed chromosome under a different contact map, such as one generated from a different resolution, restriction enzyme, or cell population, one must retrain an entirely new model. This leads to intense computational requirements when using these methods to make predictions on large data sets, such as those with high resolution. Moreover, this instance-based nature associated with traditional distance-restraint methods means that these methods tend to fail when the input data is sparse as there are fewer features that can be utilized in training.

In this paper, we present a novel distance-restraint method for 3D chromosome reconstruction from cis-chromosomal Hi-C contacts that addresses each of these limitations associated with traditional distance-restraint methods. Our method relies on a graphical interpretation of Hi-C data. From this graphical interpretation, we use a node embedding algorithm to generate features corresponding to each chromosomal locus. These features are then utilized to train a graph convolutional neural network (GCNN) to generate predictions of the xyz coordinates corresponding to each chromosomal locus.

**To the authors’ knowledge, HiC-GNN is the only chromosome structure prediction algorithm that learns models that can be stored and used to make predictions on unseen data while maintaining accuracy.** This ability to store parameters and make predictions on unseen data with accuracy is precisely our definition of generalization. Specifically, we show that our models can generalize across three data variations:

1. **Generalization across resolutions**: a model trained on the Hi-C map of a fixed chromosome at one resolution can be used to accurately predict the structure of the same fixed chromosome using a different Hi-C map resolution as the input. This allows us to train a model on low resolution data and make predictions for high-resolution data, thereby circumventing the computational expenditure associated with training a new model on high-resolution data.
2. **Generalization across restriction enzymes**: a model trained on a Hi-C map of a fixed chromosome utilizing some restriction enzyme in the Hi-C experiment can be used to accurately predict the structure of the same fixed chromosome using a Hi-C map obtained with a different restriction enzyme as an input.
3. **Generalization across cell population**: a model trained on a Hi-C map of a fixed chromosome corresponding to some cell population can be used to accurately predict the structure of the same fixed chromosome using Hi-C data obtained from a different cell population. This allows us to train a model on contact-sparse data (i.e., contact maps with fewer contact frequencies) and make predictions on denser contact maps.

These generalizations allow for several benefits associated with HiC-GNN that are absent in other methods. **Generalization 1** has the practical benefit of being able to train a model on low resolution data while still being able to make predictions on high resolution data, thereby avoiding the additional computational requirements associated with training a model on high resolution data. This is particularly important since the computational requirements of some methods limit their use to low resolution data. We show that this benefit decreases the runtime of HiC-GNN and thus yields faster results than other methods. **Generalization 2** shows that our models are robust to biases introduced by choices of restriction enzymes, i.e., we can ensure that the predicted structure of a given chromosome is consistent irrespective of which restriction enzyme was used in the training data. **Generalization 3** shows that our models are robust to contact sparsity in the data.

We validate the reconstructive performance and the generalization capabilities of our method on three separate data sets from the GM12878, GM06990, and K562 cell lines and make comparisons with four other Hi-C chromosome reconstruction methods; ShRec3D [18], ShNeigh2 [19], ChromSDE [16], and LorDG [11]. We also validate the reconstructive performance of our method using orthogonal ChIA-PET data from the GM12878 cell line.

### 1.2 Overview of other methods

There currently exist many methods for 3D chromosome reconstruction. MCMC5 is a method which uses a Markov Chain Monte Carlo (MCMC) for sampling spatial coordinates from the posterior distribution generated by interaction frequency data under a Gaussian prior [40]. BACH also uses MCMC to sample spatial coordinates, except the authors assume a Poisson distributed prior [46]. PASTIS also assumes that spatial coordinates are related to contact frequencies according to a Poisson distribution; however, spatial coordinates are optimized via maximizing the likelihood of the Poisson distribution [47]. Chromosome3D is a distance restraint method which optimizes distances using distance geometry simulated annealing [15]. LorDG is a distance restraint method that uses an objective function derived from the Lorenzian function. The Lorenzian objective smooths inconsistencies in the Hi-C due to heterogeneous cell populations by rewarding the satisfaction of consistent restraints whose value is not affected by the violation of inconsistent restraints. Finally, ChromSDE is a distance restraint method that relies on semi-definite programming to optimize the predicted structures. Moreover, ChromSDE relies on a golden search algorithm to infer the relationship between interaction frequency and distance. We chose to compare our method to LorDG and ChromSDE due to their ability to outperform several other distance and contact-based algorithms, that is use the contact data directly for 3D structure reconstruction [10], [11], [16], [23]. Thus, we can consider this methods top-performers, and representative methods for distance instance-based method for chromosome 3D structure reconstruction.

We also compare our method to ShNeigh2 and ShRec3D. Both ShNeigh2 and ShRec3D are methods that consider the neighborhood structure of contact sites in Hi-C data. Like our method, these two methods rely on a graphical interpretation of Hi-C data. This is the reason why we choose to include these two methods in our method evaluation. ShRec3D considers the neighborhood structure of contact sites by utilizing a shortest path algorithm on the Hi-C data to derive distances from contacts. The structure of the chromosome is then inferred from these distances using multi-dimensional scaling. A recently proposed method, ShNeigh [19], incorporates neighborhood dependence by defining an affinity matrix associated with the input contact matrix defined from a Gaussian distribution. The entries of this affinity matrix are then utilized as regularization terms in the objective that is thence optimized. The authors of ShNeigh present two versions of the algorithm, ShNeigh1 and ShNeigh2. The difference between these versions is that ShNeigh1 assumes a constant relationship between interaction frequency and distance, whereas ShNeigh2 optimizes this relationship dynamically. Thus, ShNeigh2 is slower than ShNeigh1 but usually produces better results. For this reason, we compared our method with ShNeigh2.

## 2 Materials and methods

The crux of our method is a graphical interpretation of the input Hi-C data. Recall that a Hi-C map for a given chromosome is an N×N symmetric matrix whose $ij^{th}$ entry corresponds to the contact frequency between locus i and locus j. N refers to the total amount of loci observed in the Hi-C map. Our method interprets this contact matrix to be an adjacency matrix corresponding to an edge-weighted, un-directed graph consisting of N nodes. In this formulation, the $ij^{th}$ entry of a given Hi-C map denotes the edge weight between node i and node j, and zero entries imply that the nodes are not connected. This graphical interpretation of the Hi-C data allows for the topology of the graph to be considered during the reconstruction.

**Fig. 1:**
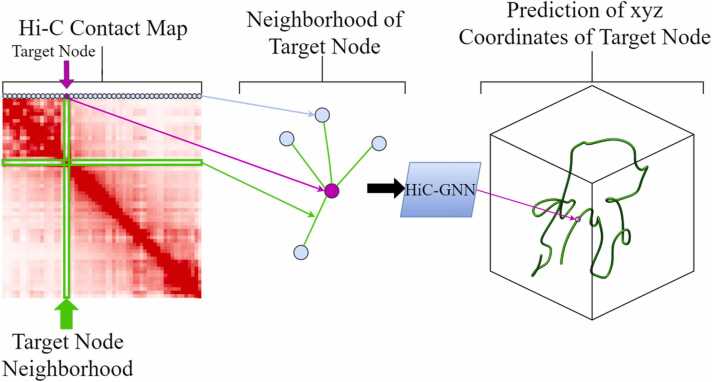
HiC-GNN 3D chromosomal structure prediction pipeline. A high-level overview of how HiC-GNN accomplishes the task of 3D chromosomal structure prediction from Hi-C data. The input Hi-C contact map is interpreted as an adjacency matrix corresponding to an edge-weighted graph. Each node within the graph corresponds to a locus in the chromosome. Given a target node, we perform graph convolutions on its one-hop neighborhood and output a predicted coordinate corresponding to the locus’ spatial position.

With this graphical interpretation, we may formulate the task of predicting the structure of the chromosome as a node regression problem. Specifically, we are given a graph with unlabeled nodes corresponding to intra-chromosomal loci and edge weights corresponding to contact frequencies between these loci. Our task is to assign xyz coordinates to each node such that the difference between the true chromosomal structure and predicted chromosomal structure is minimized. Fig. 1 gives a high-level overview of how we utilize the graphical interpretation of Hi-C data to accomplish this task.

Our method takes a Hi-C map of cis-chromosomal contacts of a given chromosome as an input. From this map, we generate feature vectors for each node using a node embedding algorithm. We also generate ground truth, or wish distances, from the input map according to a standard conversion formula typical in most distance-restraint methods. We then normalize the input Hi-C map to the range [0,1] using Knight-Ruiz (KR) matrix balancing [20] to promote numerical stability in the training process. This normalization technique also mitigates biases in the Hi-C data [21]. We use this normalized map along with the node embeddings as inputs to a GCNN. The output of the GCNN is a set of xyz coordinates corresponding to each node of the input graph. We then compute the pairwise distances between each of these coordinates and compare to the wish distances corresponding to the input Hi-C map using mean squared error (MSE). We find the optimal coordinates by minimizing MSE through backpropagation of the GCNN. This optimization is performed using the Adam optimizer [22]. We use a convergence threshold to determine when the network is sufficiently optimized, i.e., we train until MSE is below a certain value.

### 2.1 Conversion of contacts to wish distances

One challenge posed by 3D chromosome structural inference is the lack of ground truth associated with the input data. We would like to optimize the output coordinates of our model to match the true pairwise distances corresponding to the loci of the input chromosome, but these true distances are generally unknown. It has been shown both empirically and theoretically, however, that relationship between the distances and contact frequencies between two loci is inversely exponential [9], [23], [24], [25]. Thus, we can estimate the true pairwise distance between locus *i* and locus *j* by using.

$d\left(i,j\right)={\left(\frac{1}{{\mathrm{CF}}_{i,j}}\right)}^{\gamma }$where ${\mathrm{CF}}_{i,j}$, is the interaction frequency between locus *i* and locus *j*. The parameter γ is known as the conversion factor. In general, the value for γ is unknown and varies depending on the underlying chromosome. It has been shown, however, that γ lies in the range [.1,2] for most, common cell types [26]. In our experiments, we assume that the optimal conversion belongs to the set {0.1,0.2,.,2}. We train a model using ground-truth data generated for each conversion factor in this set and select the structure with the highest Spearman correlation coefficient (see the evaluation section) as the representative model. This method of converting contact frequencies to distances and generating an ensemble of structures based on multiple conversion factors is used in several other distance-restraint algorithms and has been shown to be a valid means for generating ground-truth distance data [10], [16], [27].

### 2.2 Node feature creation

Another challenge associated with our formulation of 3D structure reconstruction as a node regression problem is the lack of features associated with the nodes we would like to regress. Hi-C data only defines a graph structure through weighted edges between featureless nodes. Thus, we must create node features to serve as inputs to the regression problem. These node features ideally have two desirable properties. Firstly, we would like these node features to be correlated to the underlying graph structure, i.e., node embeddings within regions of high connectivity should be similar. Secondly, we would like this similarity defined from the graph structure to translate to similarity of node features in Euclidean space so that the 3D structure of the chromosome can be inferred from these features. A natural way to accomplish these two goals is to create vectorized representations of each node utilizing a node embedding algorithm and use these representations as the input node features.

We create node features using the LINE node embedding algorithm [28] to be input into our GCNN. LINE is a node embedding algorithm that is specifically adapted to scalable use on large graphs. We used the LINE node embedding algorithm because it has been used in previous Hi-C research and has shown success in predicting chromosome compartmentalization from Hi-C data [29]. One advantage associated with LINE in the context of this specific application is that LINE considers edge weights when generating embeddings. LINE also accounts for both first and second order proximities in the input graph. Thus, the embeddings from LINE account for correlations between the contact values of the Hi-C map and preserve higher order relationships between node neighborhoods. The general technique of LINE is as follows. Firstly, a conditional node context distribution is defined. This distribution is given by Eq. (1):

(1) $$p_{2}\left(v_{j}{|v}_{i}\right)=\frac{\exp\left(u_{j}\cdot u_{i}\right)}{\sum_{k\in N\left(v_{i}\right)}\exp\left(u_{k}^{\prime },u_{i}\right)}$$

where $v_{j}\;$are indexed nodes and $u_{i}$ are the corresponding n-dimensional, real-valued-vector feature representations. The empirical distribution p2^is then fit to $p_{2}\;$by minimizing the Kullback–Leibler (KL) divergence between these two distributions using stochastic gradient descent. Intuitively, LINE maximizes the probability of recreating the underlying graph from the computed node embeddings. The information about how to access the LINE algorithm is provided in the ‘Availability of data and materials’ section.

### 2.3 Hi-C map normalization

The inputs to the GCNN are a set of node features and the corresponding Hi-C contact map. In this context, the contact map is interpreted as an adjacency matrix corresponding to an edge-weighted graph whose weights correspond to the map’s contact frequencies. The values of these contact frequencies are often in the hundreds of thousands. Thus, to promote numerical stability of the GCNN, we normalize the input map to the interval [0,1]. We perform this normalization using Knight-Ruiz (KR) matrix balancing [20]. The result of KR balancing is a doubly stochastic matrix. This technique has been used in several other applications of Hi-C data [21].

### 2.4 Graph convolutional neural network architecture

Following the generation of node feature vectors, the regression of node xyz coordinates is performed using a GCNN. The advantage of utilizing a GCNN to estimate 3D coordinates from the input features as opposed to just using a standard neural network is two-fold. Firstly, GCNNs incorporate the graphical structure of the Hi-C data features, whereas standard neural networks have no way of interpreting graphical relationships from the data[38]. Secondly, the shape of the input layer of the network depends only on the shape of the node features and is independent of the quantity of nodes in the input adjacency matrix. This independence is what allows us to generalize models between input Hi-C maps of potentially different sizes.

Our method relies on a consolidate-update inspired by the GraphSAGE algorithm [43]. In general, the consolidate-update strategy involves a consolidation of the features of nodes in the neighborhood of a target node followed by an update of the target node's feature via some trainable function[39], [41], [42], [43]. Assume we are computing the 3D coordinates of node i with corresponding feature vector $x_{i}$ of length n. We first consolidate features of the nodes in the neighborhood of i using the Eq. (2).

(2) $$C\left(x_{i}\right)=\frac{1}{\sum_{j\in N\left(i\right)}e_{i,j}}\sum_{j\in N\left(i\right)}e_{i,j}x_{j}$$

where N(i)is the neighborhood of i and $e_{i,j}$ is the edge weight between node i and node j and $x_{j}$ is the feature vector of node j. We then compute the updated target node feature vector $x_{i}^{\prime }$ using Eq. (3).

(3) $$x_{i}^{\prime }=W_{1}x_{i}+W_{2}C\left(x_{i}\right)\;$$

where $W_{1}$ and $W_{2}$ are n×n parameter matrices. Both $W_{1}$ and $W_{2}$ are updated utilizing backpropagation. Note that, to ensure generalizability across input maps of various node quantities, $W_{1}$ and $W_{2}$ are shared across all nodes. We refer to the composition of (2), (3) as the *graph convolutional layer.* We chose to include graph convolutions in our algorithm because the convolutions allow for the predicted coordinates of a locus to be influenced by neighboring loci via the weighted aggregation of local features in Eq. (2). The weights of this aggregation are determined by the contact values between neighboring loci so that neighbors with high interaction with the target node have more influence on the corresponding predicted location of said target node. This formulation is natural because neighboring loci with high contact values have greater physical interaction with the target node.

**Fig. 2:**
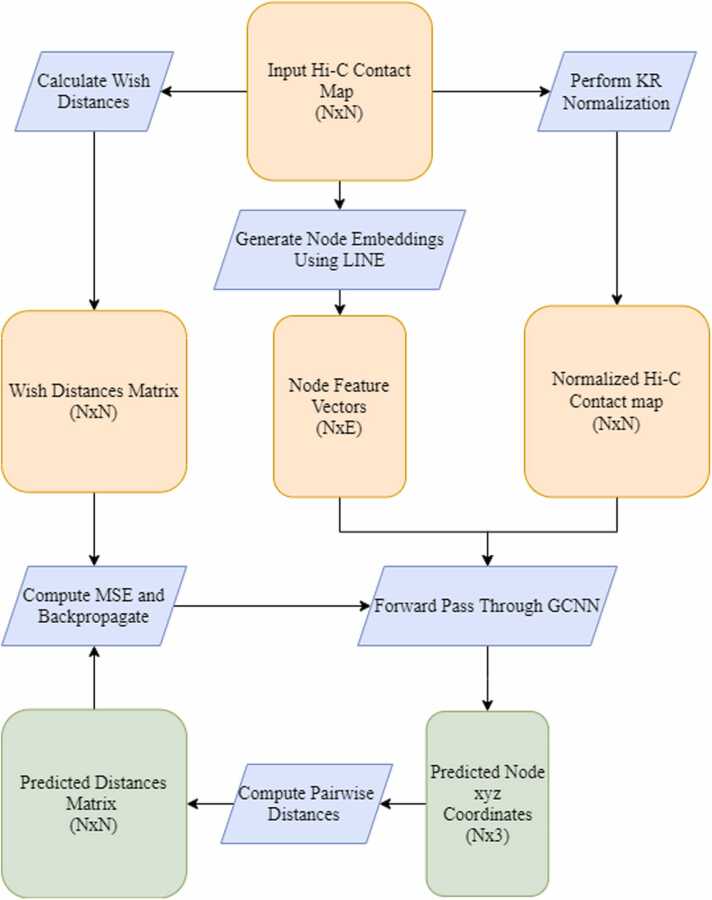
General pipeline for HiC-GNN. The pipeline for the entire HiC-GNN algorithm. From the raw input Hi-C map, we calculate wish distances using Eq. (1), we generate node embeddings using the LINE algorithm, and we compute a normalized map using KR normalization. The node feature vectors and the normalized Hi-C map are then used as inputs to the graph neural network. The graph neural network is optimized by minimizing the MSE of the pairwise distances of the output structure to the wish distances. Here, N refers to the number of loci and E refers to the size of the embeddings.

**Fig. 3:**
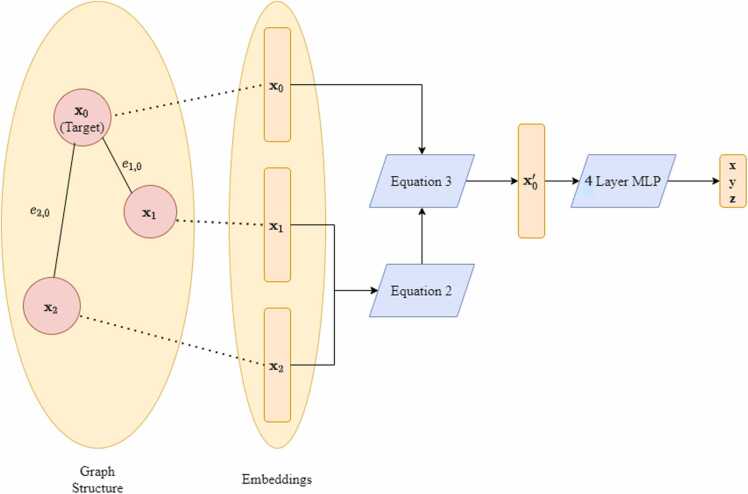
Architecture of the GCNN. This figure details the architecture of the GCNN. The node features of the target node’s neighborhood are consolidated using Eq. 2. The representation of the target node is then updated using Eq. 3. These two equations define the graph convolutional layer. Finally, the coordinates of the target node are predicted using a 4-layer MLP. Here, we are predicting the xyz coordinate of $x_{0}$, where $x_{1}$ and $x_{2}$ are the neighbors of $x_{0}\;$with edge weights of $e_{1,0\;}$and $e_{2,0}\;$respectively.

Following the graph convolutional layer, the updated node features following a single graph convolutional layer are then passed through a four-layer multilayer perceptron (MLP) which outputs the xyz coordinates corresponding to the target node. The parameters of the MLP are shared across all nodes. Each hidden layer of the GCNN is followed by a ReLU activation. The output layer is not followed by any activation to not restrict the domain of the predicted structure. We then compute the pairwise distances between each of the output xyz coordinates and compare these output distances to the wish distances using mean squared error (MSE). We then optimize the parameters of the network utilizing backpropagation and the Adam optimizer [22] to minimize the MSE between the distances corresponding to the output structure and the wish distances. We use a convergence threshold to determine when the network is sufficiently optimized, i.e., we train until MSE is below a certain value. The entire HiC-GNN algorithm can be visualized in Fig. 2. The architecture of the GCNN can be visualized in Fig. 3.

### 2.5 Embedding alignment for generalization

The process of generalizing the results of HiC-GNN involves training the GCNN on a Hi-C map and its corresponding embeddings from one set of data and utilizing this trained network to generate structures using the embeddings and maps of another set of data. It is possible, however, that the embedding distributions vary significantly across different data, thereby making generalization difficult. Thus, we assume that embeddings are only approximately similar up to isometry, i.e., we assume that the embeddings between two separate chromosomes are approximately equivalent up to rotation, translation, and scaling irrespective of the restriction enzyme, cell population, and resolution of the maps. To test this assumption, we employ an embedding realignment procedure prior to testing a generalized model on new embeddings[44].

Assume we have two N×E embeddings matrices, A and B. Here, N refers to the number of chromosomal loci and E refers to the embedding size. We would like to find a linear transformation that minimizes the Euclidean distance between A and B. Formally, we would like to compute (4).

(4) $$T\;=\;arg\;min_{\Omega }||\;\Omega A\;-\;B\;|{\;|}_{F}$$

$\;||\cdot |{|}_{F}$ denotes the Frobenius norm. This problem is known as the generalized Procrustes problem (GPP) [30]. Computing the matrix T in the GPP is equivalent to computing the singular value decomposition of the matrix $\Omega =BA^{T}$ [31]. Thus, the task of embedding realignment has a closed form solution and requires no additional training. Note that, in our applications, it is not guaranteed that the embedding matrices A and B have the same size due to differing numbers of chromosomal loci across differing resolutions. For this reason, we employ a simple expansion procedure to match the number of rows in the embedding matrices which we describe below:

#### 2.5.1 Expansion procedure for feature alignment

The alignment procedure used in our model generalization assumes the existence of a linear transformation between the embedding spaces of two distinct Hi-C maps. In the case of generalizing across resolutions, however, we run into the issue of the dimensions of these spaces differing. Specifically, if A denotes the embedding matrix corresponding to the lower resolution data and B denotes the embedding matrix corresponding to the higher resolution data, then ΩA−Bis not well defined since the number of rows in A is less than the number of rows in B. We fix this problem using the following expansion procedure.

Assume we are performing a resolution generalization of a given chromosome. In our experiments, A always corresponds to the map at 1 mb resolution and B either corresponds to the map at 500 kb or 250 kb resolution. This implies that B either as twice or four times the number of rows of A. See Table 1 for a visual representation of why this is the case.

**Table 1:** Table showing which rows of the embedding matrices correspond to which interaction sites.

Row	1 mb Loci	500 kb Loci	250 kb Loci
0	0 – 1	0 – 0.5	0 – 0.25
1	1 – 2	0.5 – 1	0.25 – 0.5
2	2 – 3	1 – 1.5	0.5 – 0.75
3	3 – 4	1.5 – 2	0.75 – 1
…	…	…	…

The row column represents the row of an arbitrary embedding matrix. The loci columns depict which interaction sites the row of the embedding matrix corresponds to at a given input resolution. These values are given in millions of base pairs. For example, embedding of the first row of an embedding matrix generated from 1 mb data corresponds to the portion of the chromosome between base pair 0 and base pair 1,000,000. The same row corresponds to the portion of the chromosome between base pair 0 and base pair 500,000 for an embedding matrix generated from 500 kb data, and base pair 0 and base pair 250,000 for an embedding matrix generated from 250 kb data. To force the embeddings matrices to have the same number of rows, we simply repeat additional rows of the 1 mb data such that the chromosomal region of the equivalent rows in the higher resolution embeddings matrix is contained in the chromosomal region of the given row in the 1 mb embeddings matrix. See Table 2, Table 3 for an example of this expansion procedure applied to the 500 kb case and the 250 kb case. By expanding the 1 mb embeddings matrix in this way, we ensure that the dimensions of matrix A and matrix B match in the alignment procedure. Moreover, we ensure that the corresponding rows between these two matrices come from the same regions in the chromosome.

**Table 2:** Table showing how we expand the 1 mb embeddings matrix to match the shape of the 500 kb embeddings matrix.

Row	Expanded 1 mb Loci	500 kb Loci
0	0 – 1	0 – 0.5
1	0 – 1	0.5 – 1
2	1 – 2	1 – 1.5
3	1 – 2	1.5 – 2
…	…	…

**Table 3:** Table showing how we expand the 1 mb embeddings matrix to match the shape of the 250 kb embeddings matrix.

Row	Expanded 1 mb Loci	250 kb Loci
0	0 – 1	0 – 0.25
1	0 – 1	0.25 – 0.5
2	0 – 1	0.5 – 0.75
3	0 – 1	0.75 – 1
…	…	…

Note that the alignment process for Hi-C data often yields regions with no contacts. For sake of reducing the size of these data, many Hi-C maps simply do not include these contacts. In order to circumvent this issue, we include zero contacts in this expansion procedure so that it is guaranteed the number of loci for higher resolution is a scalar multiple of the number of loci for the lower resolution.

### 2.6 Hyperparameter optimization

Prior to generating results on real Hi-C data, we tuned the hyperparameters of HiC-GNN by performing a grid search on the simulated Hi-C data from Trussart et al. [27]. The Trussart et al. dataset consists of multiple Hi-C maps generated from the simulation of the Hi-C protocol on multiple worm-like chain (WLC) chromosome models at varying levels of noise and structural variability. The advantage of using simulated data for hyper-parameter tuning is that unlike in the case of real Hi-C data, the structure of the chromosome is known, thereby allowing us to make a direct comparison between the outputs of HiC-GNN and the true distances of the chromosome. By optimizing the hyper-parameters of our model in a setting in which the outputs can be compared with a known structure, we ensure that our model will perform well on data where the true structure of the input chromosomes is unknown as well.

We performed our experiments on a simulated chromosome of minimal structural variability with a corresponding simulated Hi-C map involving zero noise. Specifically, we used the maps corresponding to group 0 of structural variability with ∝=50 as the noise parameter within the Trussart et al. study. We chose this chromosome-noise configuration so that the optimal parameters selected by the grid search were not influenced by randomness associated with high levels of structural variability or noise. In our grid search, we aimed to optimize the node embedding size, the sizes of the hidden layers of the GCNN, the learning rate, and the convergence threshold. The results of this grid search can be found in Table 4, Table 5. The optimal parameters are shown in bold. A spreadsheet containing all of the dSCC values for the different configurations for hyperparameter tuning can be found in the Additional file 1.

**Table 4:** Optimal layer sizes as determined by the grid search on the simulated data.

Embeddings Size	1024	**512**	256
GC Layer	1024	**512**	256
MLP Layer 1	512	**256**	128
MLP Layer 2	256	**128**	64
MLP Layer 3	128	**64**	32
MLP Output	**3**	-	-

Note that the MLP must have an output of size 3 to correspond to the xyz coordinates of the chromosomal loci. The selected network settings based on the grid search are in bold on the table.

**Table 5:** Optimal learning rate and convergence threshold as determined by the grid search on the simulated data.

Learning Rate	0.1	0.01	**0.001**	0.0001
Convergence Threshold	$10^{-2}$	$10^{-4}$	$10^{-5}$	$10^{-12}$

We explored different learning rate and convergence thresholds; the selected network settings based on the grid search are in bold on the table.

### 2.7 Evaluation

To validate the reconstructive accuracy of our method, we use distance Spearman Correlation Coefficient (dSCC). dSCC is a non-parametric measure of rank correlation. general, dSCC values closer to 1 imply higher reconstructive accuracy. The formula for dSCC is given by Eq. (5).

(5) $$dSCC=\frac{\sum_{i\in D^{\prime }}\left(X_{i}-\bar{X}\right)\sum_{i\in D}\left(Y_{i}-\bar{Y}\right)}{\sqrt{\sum_{i\in D}{\left(X_{i}-\bar{X}\right)}^{2}\sum_{i\in D}{\left(Y_{i}-\bar{Y}\right)}^{2}}}\;$$

$D^{\prime }$ is the set of pairwise distances between all loci of the generated model, $X_{i}$ is the rank of distance i in $D^{\prime }$, D is the set of wish distances corresponding to the input contact frequencies of the chromosome, and $Y_{i}$ is the rank of wish distance i in D. $\bar{X}$, $\bar{Y}$ are the mean of their corresponding ranked vectors in $D^{\prime }$ and D respectively.

Note that dSCC is a non-parametric measure of rank correlation. The advantage to evaluating reconstructive performance using a ranked measure of similarity is that, unlike mean-squared error, the measure is scale invariant. Intuitively, the model may output a perfect match of the chromosome, but the xyz coordinates may be scaled by a constant. This scaling would be accounted for in a non-ranked measure of correlation and would likely decrease the correlation value. This decrease in correlation would falsely imply that the generated model is inaccurate when the only dissimilarity between it and the ground truth is the scale and location in space. Since the purpose of modeling the chromosome in 3D space is solely for visualization, the scale of the output should not matter. Thus, dSCC is an appropriate measure of structural similarity in this context. Based on the work of Trussart et al. [27], the dSCC of the output structure with the wish distances from the conversion in equation (0) serve as a good proxy for structural similarity to the true, unknown structure of the chromosome. Thus, in general, it is unnecessary to evaluate the dSCC using orthogonal data. Since dSCC are dependent on the conversion used during model training, however, we also evaluate our method using orthogonal ChIA-PET and FISH data in order to validate the use of these metrics as a proxy for structural similarity to the true chromosome structure.

## 3 Data

### 3.1 Real Hi-C data

To test the reconstructive performance of HiC-GNN, we utilized three data sets consisting of real Hi-C data. The first data set corresponds to the human GM12878 cell line from Rao et al. [32]. This data set consists of the Hi-C maps of 23 chromosomes generated from the Mbol restriction enzyme at 1 mb, 500 kb, and 250 kb resolutions. This data set was downloaded from the Genome Structure Database (GSDB) repository [33] under the GSDB ID: OO7429SF. We utilized this data set to test Generalization 1. The second data set corresponds to the human GM06990 cell line from Lieberman et al. [9]. This data set consists of the Hi-C maps of 22 chromosomes generated from the Ncol and *Hin*dIII restriction enzymes at 1 mb resolution. We utilized this data set to test Generalization 2. The third data set corresponds to the human K562 cell line from Rao et al. [32]. This data set consists of several Hi-C maps of 23 chromosomes generated from the Mbol restriction enzyme at 1 mb resolution. The genome-wide maps of this data set vary in their total number of contacts, ranging from 53 million to 932 million. This data set was downloaded from the Juicebox tool developed by Durand et al. [34]. We utilized this data set to test generalization 3.

### 3.2 ChIA-PET data

Chromatin immunoprecipitation (ChIP) is a technique to investigate protein specific interactions in chromosomes. ChIP relies on antibodies to precipitate specific proteins, histones, or transcription factors from cell populations. ChIP can also be combined with sequencing technologies to quantify these interactions [35]. Chromosome Interaction Analysis by Paired-End Sequencing (ChIA-PET) [36] is an example of such a technology. The main difference between ChiA-PET and Hi-C data is that the ChiA-PET technique measures interactions associated with a unique protein in the chromosome, whereas the Hi-C technique measures interactions between any loci in the chromosome.

To further validate our results on the real Hi-C data, we compare the outputs of our method when using Hi-C data to the interaction frequencies of an orthogonal ChiA-PET data set. We performed this validation using ChIA-PET data from the NCBI GEO database (GEO accession: GSE72816) for the RNAPII ChIA-PET data from human GM12878 cells [37]. This data measures interactions between the RNA polymerase II multicomplex; a protein complex that is responsible for gene transcription.

### 3.3 FISH data

Fluorescent in situ hybridization (FISH) is a technique in which specific DNA fragments are colored using fluorescent dye and are then attached to a chromosome using in situ hybridization. The presence of this flouresent dye allows for direct observation and measurement of distances in the chromosomes using microscopes. We further validated our method using the FISH data provided by Rao et al. [32]. This particular FISH data measures the distance between three peaks called from the Hi-C maps of chromosomes 11, 13, 14, and 17 of the GM12878 cell line.

## 4 Results

### 4.1 GM12878 cell line dataset

#### 4.1.1 Generalization 1: generalization across input resolution

To test the reconstructive performance of HiC-GNN on real data, we evaluated the distance Spearman Correlation Coefficient (dSCC) of outputs when evaluated on Hi-C maps from the GM12878 cell line generated with Mbol restriction enzyme. To test for the effects of variability in resolution, we generated models on three separate resolutions: 1 mb, 500 kb, and 250 kb. We compared the dSCC of our output models to the dSCC of the output models of the four other methods using the optimal hyper-parameters suggested by the authors of both methods.

We also utilized the GM12878 cell line to test how well HiC-GNN can generalize across input resolutions. To do this, we generated embeddings for one chromosome at 1 mb, 500 kb, and 250 kb resolutions. We then trained our GCNN using the contact maps and corresponding embeddings of the 1 mb data until convergence is met and stored the optimal conversion factor. Following this training, we aligned the embeddings of the 500 kb and 250 kb data to those of the 1 mb data. We then generated structures using these aligned embeddings and their corresponding Hi-C contact maps as inputs to the pre-trained GCNN. Finally, we calculated the dSCC between the pairwise distances of the generated structures and the wish distances calculated from the input contact maps. Note that, since dSCC does not depend on the conversion factor, we simply used a conversion value of 1 for each calculation.

**Fig. 4:**
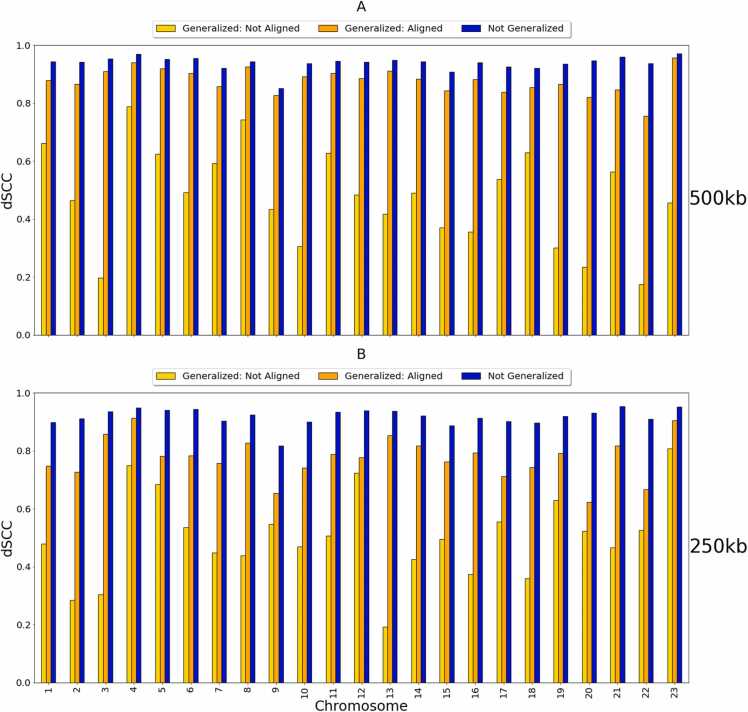
dSCC comparison: generalized and non-generalized models at 500 kb (A) and 250 kb (B) resolution. The figure shows the dSCC values for generalized and non-generalized HiC-GNN models at 500 kb and 250 kb resolution both with and without aligned node embeddings. The generalized models were trained on 1 mb data. The difference in dSCC values between the aligned and non-aligned embeddings implies that the alignment procedure has a positive effect on reconstructive performance. The high dSCC values of the generalized models also imply that the HiC-GNN models can generalize to higher resolution data.

Fig. 4 shows a comparison between the output dSCC values of the generalized HiC-GNN models and the output dSCC values of the non-generalized HiC-GNN models on the 500 kb and 250 kb data. By generalized models, we mean models that were trained on the 1 mb data and tested on the higher resolutions data. By non-generalized models, we mean models that were trained and tested on data of the same resolution. In these figures, we also include the output dSCC of the generalized HiC-GNN models using un-aligned embeddings as inputs to show the effect of the alignment procedure on reconstructive performance. From these figures, two things are clear. Firstly, the embedding alignment procedure increases the reconstructive performance of HiC-GNN. This suggests that the assumption of approximate similarity up to isometry of node embeddings is valid. Secondly, although there is some decrease in dSCC associated with the generalized models, most of the values are above 0.8 for the 500 kb generalization and above 0.7 for the 250 kb generalization. This suggests that HiC-GNN is indeed generalizing to these higher resolution data.

**Fig. 5:**
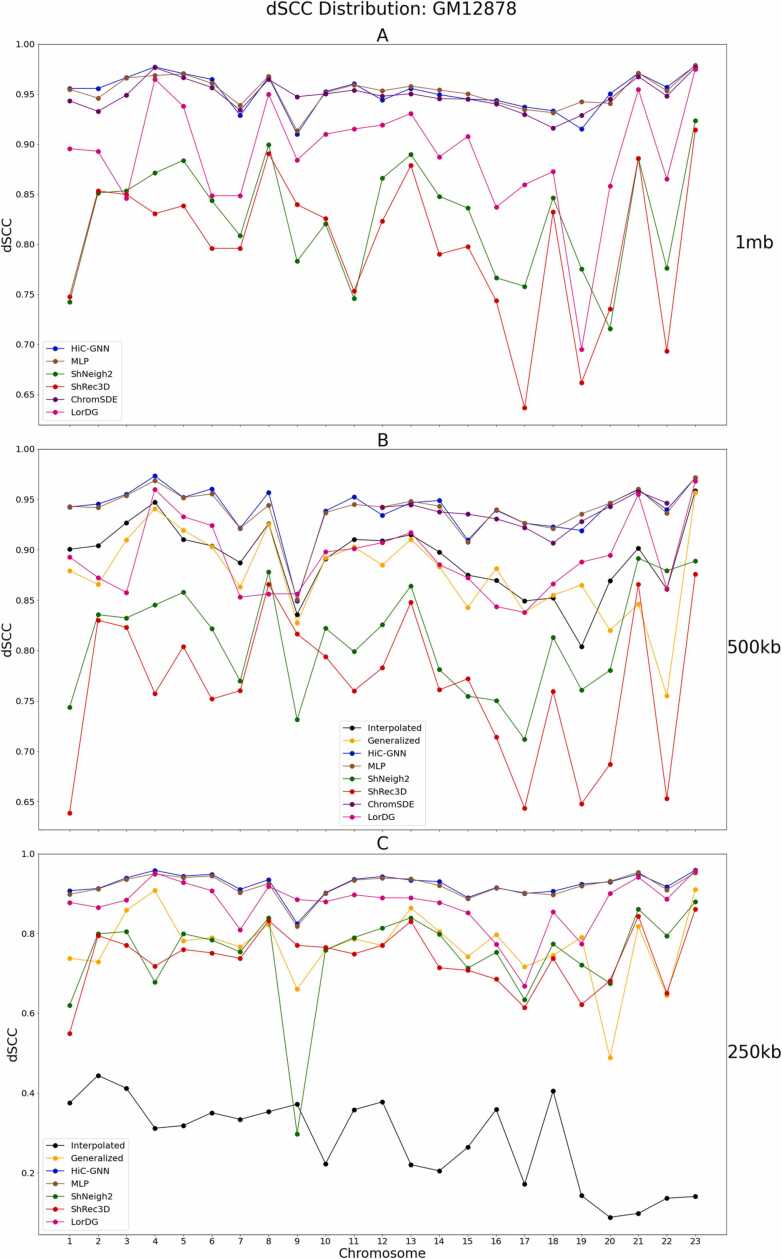
dSCC comparison: 1 mb (A), 500 kb (B), 250 kb (C) resolution. The figure shows a comparison of HiC-GNN with the other methods on the 1 mb (A), 500 kb (B), and 250 kb (C) GM12878 data using dSCC. HiC-GNN is either on-par or outperforms the other methods on the majority of the chromosomes.

**Fig. 6:**
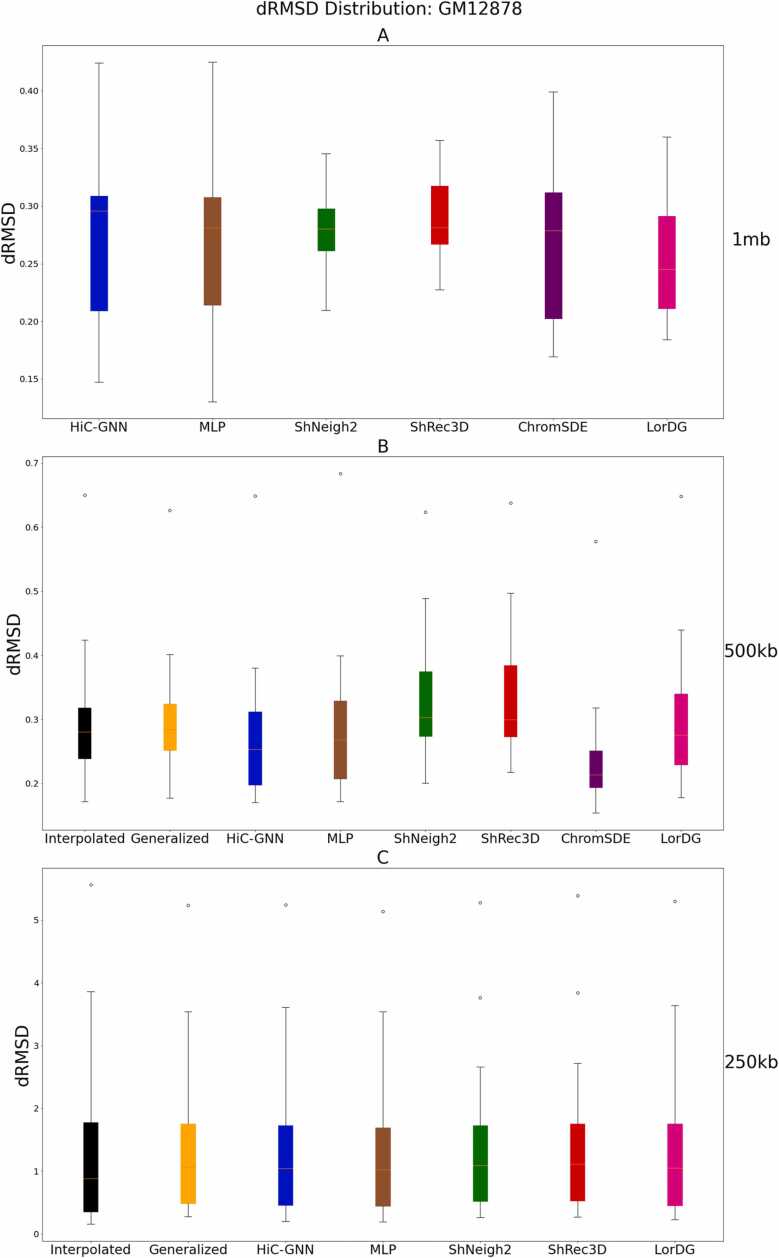
dRMSD comparison: 1 mb (A), 500 kb (B), 250 kb (C) resolution. The figure shows a comparison of HiC-GNN with the other methods on the 1 mb (A), 500 kb (B), and 250 kb (C) GM12878 data using dRMSD.

Fig. 5, Fig. 6 show the dSCC and distance root mean squared error (dRMSD) comparison of HiC-GNN with the four other methods on 1 mb, 500 kb, and 250 kb data. The dRMSD is the root mean squared error between the pairwise distances of the optimized structure and the wish distances of the contact map. To ensure a fair comparison, we computed the dRMSD using the optimal conversion factor found by each respective method. Moreover, since dRMSD is sensitive to the scale of the structure, we re-scaled all structures by minimizing their Euclidean distance from the HiC-GNN structures using Procrustes analysis.

Note that there are several missing data points for ChromSDE on the 500 kb and 250 kb data due to computational restraints associated with running the algorithm on these larger data sets. We also included the following baselines in this comparison. To test whether the graph convolutions contribute to structural accuracy, we also generated structures using the same embeddings for HiC-GNN but with a simple 4-layer MLP with no graph convolutions.

To test whether the generalized models are indeed generalizing, we compared with a linear interpolation of the 1 mb structures. For each chromosome, the interpolated structure for 500 kb was found by adding a single coordinate on the line connecting each coordinate in the 1 mb HiC-GNN structure. The same procedure was used to generate the interpolated structures at 250 kb resolution by interpolating three points instead of one. Note that this interpolation procedure results in structures with the same spatial configuration of the 1 mb outputs only with more points so that their dSCC and dRMSD may be compared with the higher resolution maps.

**Fig. 7:**
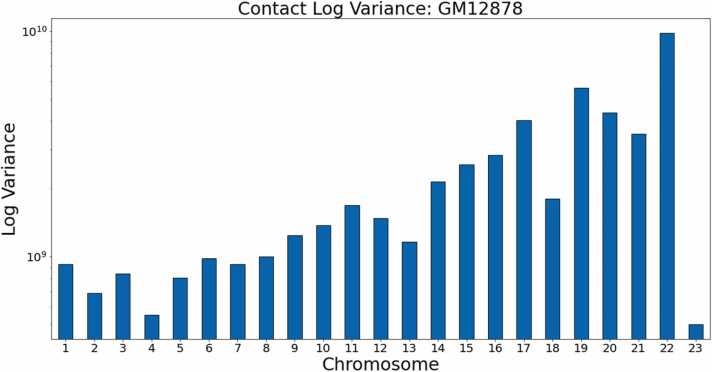
Contact variances: GM12878 data. The figure shows the distribution of log-variances of contacts for each chromosome.

From these figures, it is clear that the non-generalized HiC-GNN either outperforms or is on par with the other methods for dSCC. Moreover, the generalized HiC-GNN models either outperform or are on par with ShRec3D and ShNeigh at 500 kb despite being trained on half as many data instance. Also, although the interpolated structures are competitive with the generalized structure on 500 kb, the generalized structures perform significantly better at 250 kb. It is also worth noting that the dSCC values of HiC-GNN have less variation than most of the other methods. One of the main causes of variation in the dSCC values is high variance in the contact data. Fig. 7 shows the variance in the contacts for each chromosome in this data set. Note that the dSCC values of chromosomes with high contact variance are significantly reduced for most of the other methods, particularly on chromosome 22. This shows that HiC-GNN is also more robust to variance in the underlying contact data.

**Fig. 8:**
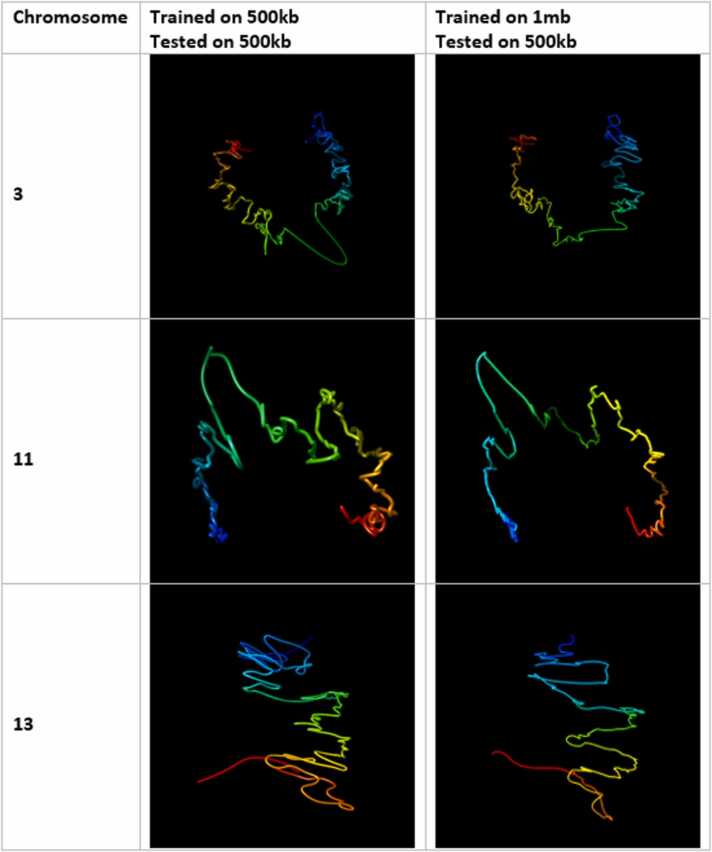
Visual comparison of structures generated from HiC-GNN generalized across resolution at 500 kb. The first column lists the chromosomes for which the 3D structure prediction was done, the second column shows the structures generated from a model trained and tested on a 500 kb map and the third column shows structures generated from a model trained on a 1 mb map and tested on a 500 kb map.

**Fig. 9:**
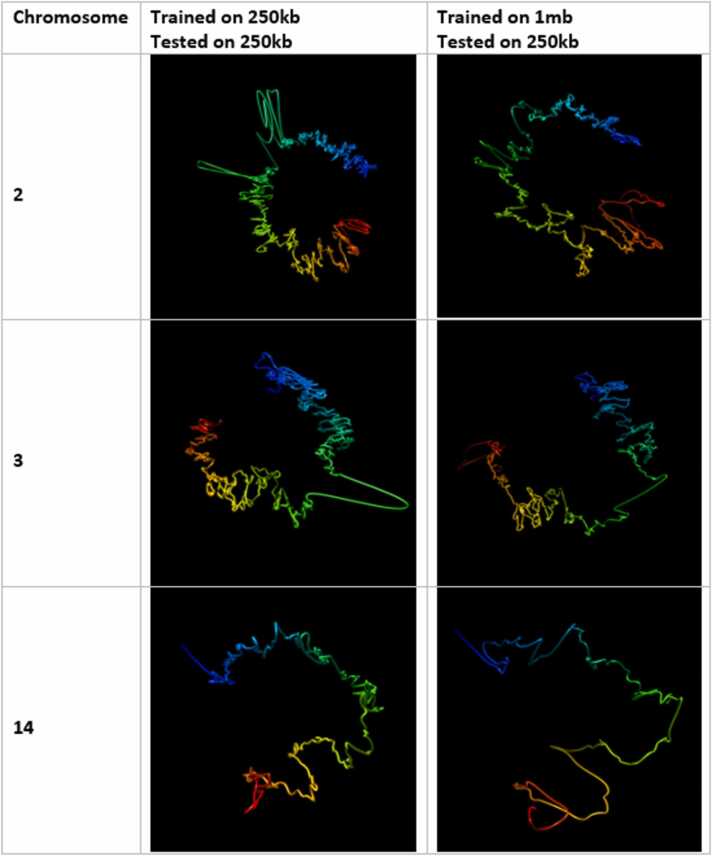
Visual comparison of structures generated from HiC-GNN generalized across resolution at 250 kb. The first column lists the chromosomes for which the 3D structure prediction was done, the second column shows the structures generated from a model trained and tested on a 500 kb map and the third column shows structures generated from a model trained on a 1 mb map and tested on a 500 kb map.

Fig. 8, Fig. 9 show the output structures of HiC-GNN corresponding to the generalized models and the original models for chromosomes 3, 11, and 13 and 2, 3, and 14 respectively. One can see that the generalized structures are indeed qualitatively similar to the non-generalized structures.

### 4.2 Validation on ChIA-PET data

Note that the results from Fig. 4, Fig. 5, Fig. 6 imply a high correlation between the output model and the input wish distances. Thus, we know our method can accurately estimate 3D coordinates from a set of wish distances. Trussart et al.[27] showed that the dSCC between the distances corresponding to output models and the wish distances of the Hi-C map is a good proxy for model accuracy. Before discussing additional results involving dSCC, however, we further validate that our method does indeed produce representative models by comparing the results generated on the GM12878 cell line with orthogonal ChIA-PET data.

The ChIA-PET data provided by [37] consists of contact maps measuring interactions between the RNAPII complex in all 23 chromosomes of the GM12878 cell line. From these contact maps, RNAPII loops were identified by considering contact regions that have an interaction frequency greater than or equal to 5. To validate our method, we split this ChIA-PET data into two sets: one containing looped regions and one containing non-looped regions. We then calculated the distances of our output models between the identified looped and non-looped regions separately for each chromosome. If our models are representative of the true structure of the chromosome, then distances corresponding to looped regions of our output models should typically be smaller than distances corresponding to non-looped regions.

**Fig. 10:**
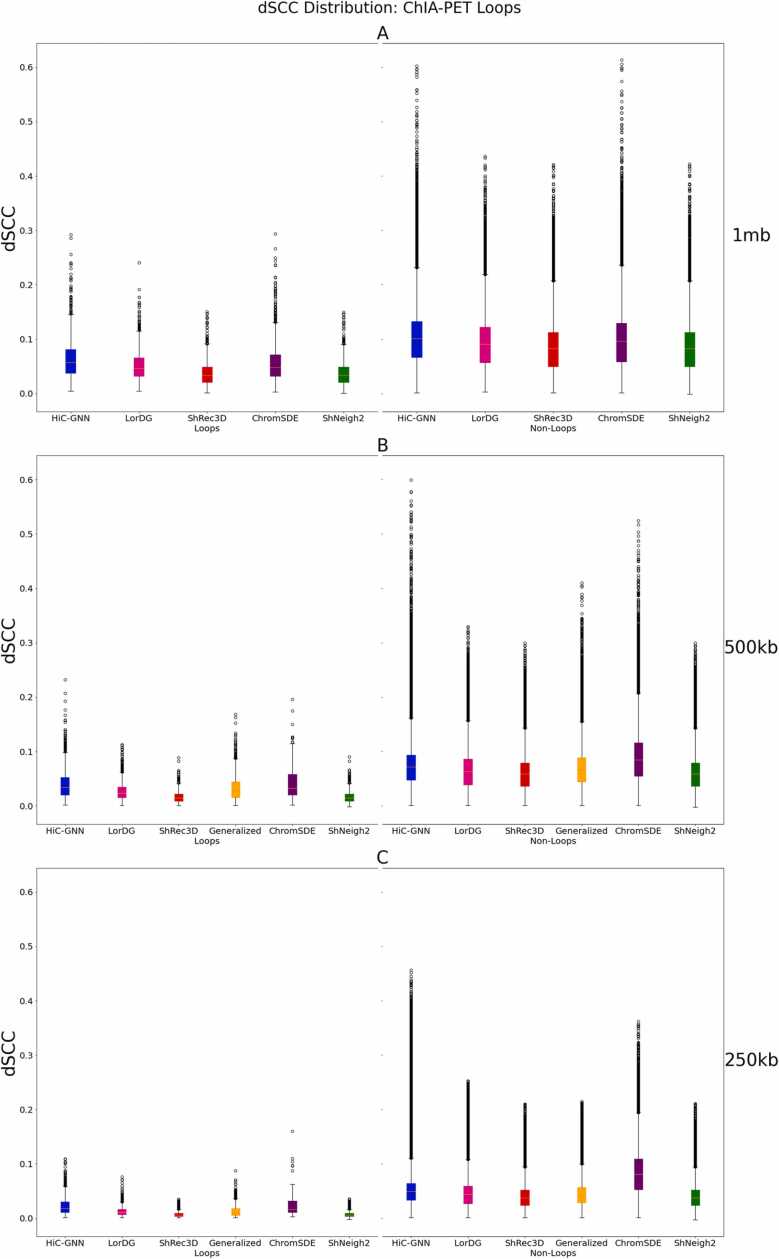
Comparison of distances for looped and non-looped regions on GM12878 across all chromosomes at 1 mb (A), 500 kb (B), and 250 kb (C) resolutions. The figure shows the box plots for the looped and non-looped regions for all chromosomes combined in the GM12878 cell line for generalized and non-generalized HiC-GNN models at 1 mb (A), 500 kb (B), and 250 kb (C) resolutions along with all other methods.

Fig. 10 shows the box plots for the looped and non-looped regions for all chromosomes combined for structures generated from both non-generalized and generalized HiC-GNN models at 1 mb, 500 kb, and 250 kb resolution. We also included in these figures the same distributions of distances for the other methods included in our comparison. From these figures, it is clear that the distribution of distances corresponding to the looped regions is centered around smaller values, thereby implying that the outputs of our method are consistent with the true structure of the chromosomes. This is true for both the generalized and non-generalized models.

Note that, although some methods have a smaller distribution of distances for the looped regions and a larger distribution of distances for the non-looped regions, this does not necessarily imply that the reconstructive accuracy of these methods is higher than HiC-GNN. The metric that matters in this test is not necessarily the mean values for these distributions, but rather that the mean values for the looped regions is smaller than that of the non-looped regions. We found that each method, including HiC-GNN, has a significantly smaller (p = 0.001) mean for the looped regions.

### 4.3 A/B compartmentalization

It is known that human chromosomes tend to organize into two primary compartments known as the A compartment and the B compartment. These compartments loci that belong to the same compartment generally have lower pairwise distances than loci that belong to different compartments. Thus, we should expect there to be a significant difference between the means of inter and intra-compartmental distances for our output structures. To validate this hypothesis, we first identified the A and B compartments for each chromosome in GM12878. These compartments were identified by separating the positive and negative entries of the principal eigenvector corresponding to the Pearson correlation matrix of a normalized contact map per the procedure presented by Lieberman et al. [9]. Loci corresponding to positive entries in this first principal eigenvector belong to compartment A and loci corresponding to negative entries in the first principal component belong to compartment B. We then measured the pairwise distances of all loci that are intra-A, all loci that are intra-B, and all loci that are A-inter-B (i.e., one belonging to A and the other belonging to B) for each output structure for HiC-GNN.

**Fig. 11:**
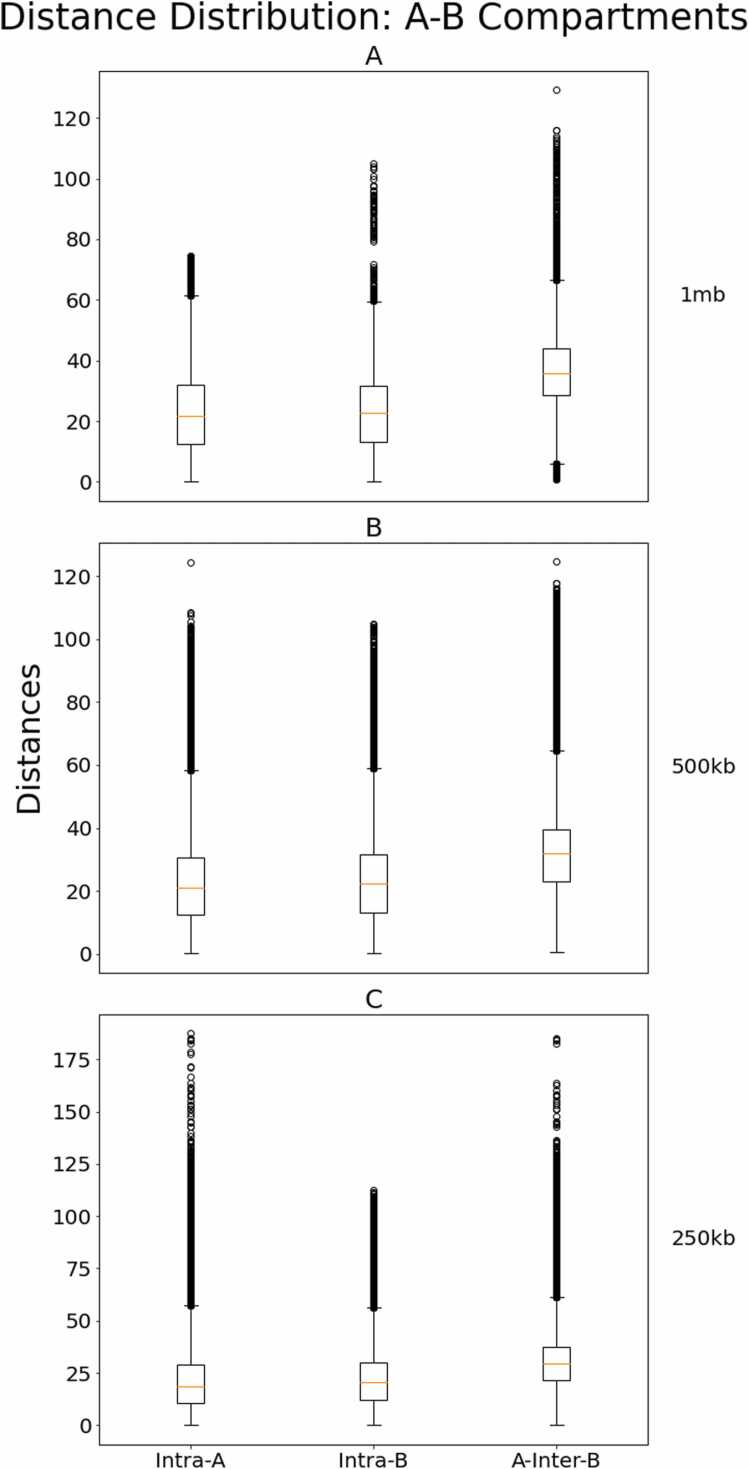
Comparison of distances for intra and inter-compartmental regions on GM12878 across all chromosomes at 1 mb (A), 500 kb (B), and 250 kb (C) resolutions. The figure shows the box plots for the intra-A, intra-B, and A-inter-B regions for all chromosomes combined in the GM12878 cell line for HiC-GNN models at 1 mb (A), 500 kb (B), and 250 kb (C) resolutions.

**Fig. 12:**
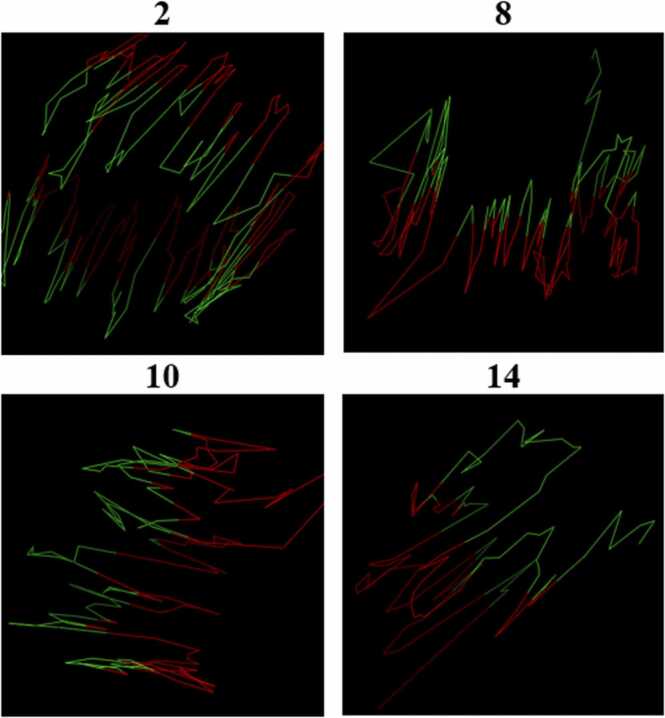
Qualitative comparison of structures with A/B compartments for GM12878 at 1 mb resolutions. The figure shows the output structures with the A (red) and B (green) compartments color-coded for chromosomes 2, 8, 10, and 14 at 1 mb resolution. Clearly, there is a divide between the two compartments in the output structures.

Fig. 11 shows the distribution of distances for each of these three distance subsets for each chromosome at all three resolutions. From this figure, it is clear that the average intra-distance (left and middle boxes) is lower than the average inter-distance (rightmost box), thereby validating that our structures organize into well-defined A-B compartments. Moreover, this difference between the means is statistically significant (p = 0.001). Fig. 12 also shows the color-coded A/B compartments for 4 randomly selected chromosomes at 1 mb resolution for a qualitative validation of this phenomenon.

### 4.4 Validation on FISH data

FISH data includes the true, measured distances between loci on a chromosome. Thus, we may compare the FISH distances with the distances corresponding to our output models to validate that our method is indeed producing results that are consistent with the true structure of the chromosome. We used the FISH distance data from Rao et al. [32], which measured two peaked regions, denoted by L1 and L2, for chromosomes 11, 13, 14, and 17. These looped peaked regions were identified by the authors of this study using their HiCCUPS loop detection algorithm. A third, non-peak region, L3, was also included in the study as a control. For chromosomes 11, 13, 14, and 17, the FISH distances between L1 and L2 was shorter than that between L2 and L3.

To further validate HiC-GNN, we identified these regions on chromosomes 11, 13, 14, and 17 for our output structures at 250 kb resolution. We then computed the L1-L2 and L2-L3 distances for our output structures in order to check that the same pattern persists as in the FISH data. We choose models at 250 kb resolution because models at 1 mb and 500 kb resolution do not have enough fidelity to pinpoint the L-regions to the same degree of accuracy in the FISH study. Table 6 shows the L-region distances for each chromosome along with the corresponding contact probabilities from the respective Hi-C maps. Clearly, the L1-L2 distances are smaller than the L2-L3 distances as desired. Moreover, the distances inversely match the trend between the contact probabilities as one would expect from the inverse relationship between contact probability and distance.

**Table 6:** FISH data validation result on GM12878 chromosomes 11, 14, 13, and 17 at 250 kb resolution.

Chromosome	L1-L2 Distance	L1-L2 Probability	L2-L3 Distance	L2-L3 Probability
11	3.3	1.49 × 10^−4^	3.7	1.35 × 10^−4^
14	6.1	6.97 × 10^−5^	11	3.51 × 10^−5^
13	1.9	2.72 × 10^−4^	3.3	1.12 × 10^−4^
17	1.8	2.72 × 10^−4^	9	1.13 × 10^−4^

The table shows the L1-L2 distance and L2-L3 distance for chromosomes 11, 14, 13, and 17 at 250 kb resolution. The table also shows the contact probabilities for these regions. The FISH data provided by Rao et al. [32] shows that the L1-L2 distance should be less than the L2-L3 distance. The table shows that this is indeed the case.

### 4.5 Runtime comparison

To show the practical benefit of generalization 1, we measured the runtime of HiC-GNN for models generalized across resolution. We compare these runtimes to those of LorDG in this experiment since LorDG is the fastest of all other methods considered in this paper. Thus, for visual simplicity, our figures only show the runtimes of LorDG and HiC-GNN. For this experiment, we selected 11 Hi-C maps increasing in the number of loci from the GM12878 data set. The maps containing less than 600 loci were all 1 mb in resolution. The maps containing more than 600 loci were either 500 kb or 250 kb in resolution. We measured the runtime of LorDG along with the training and inference time of HiC-GNN. For the maps containing more than 600 loci, we also measured the training time for the same map at 1 mb resolution. Note that we included the grid-search for the conversion factor in our measurements of the runtime. for HiC-GNN.

**Fig. 13:**
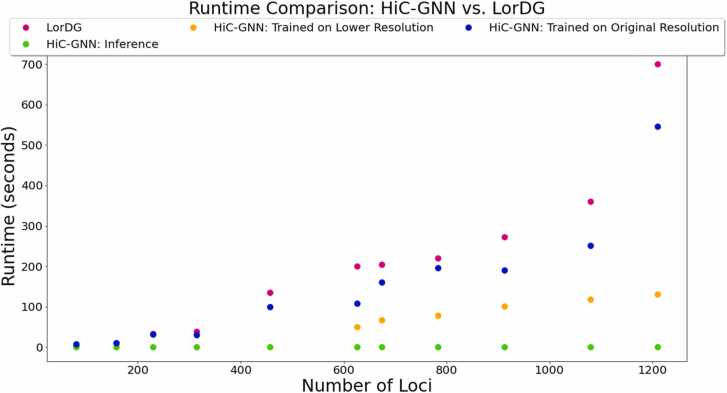
Runtime comparison of HiC-GNN to LorDG. The figure compares the runtime of each method for contact maps of increasing number of loci. For contact maps with greater than 600 loci, we trained HiC-GNN on the corresponding 1 mb resolution map. All inference was run on the original resolution. The orange dots can be interpreted as the runtime of generalized HiC-GNN models for generalization 1.

Fig. 13 shows the results of this comparison. Even the HiC-GNN models that were trained on the full resolution have a faster runtime than LorDG. This difference is even greater, however, for the models that were trained on the lower resolution. In fact, the results generated on 1200 loci map had a runtime of less than a fifth of that of LorDG. It is important to note that even though we trained HiC-GNN on 1 mb resolution maps, the inference was run on the corresponding higher resolution map (either 500 kb or 250 kb depending on the number of contacts on the x-axis). Thus, the resulting structures produced by HiC-GNN are of the same resolution of LorDG, but they were generated in a fraction of the time. This is precisely the practical benefit of generalization 1- low resolution training times with high resolution outputs.

### 4.6 GM06990 cell line dataset

#### 4.6.1 Generalization 2: generalization across restriction enzyme

**Fig. 14:**
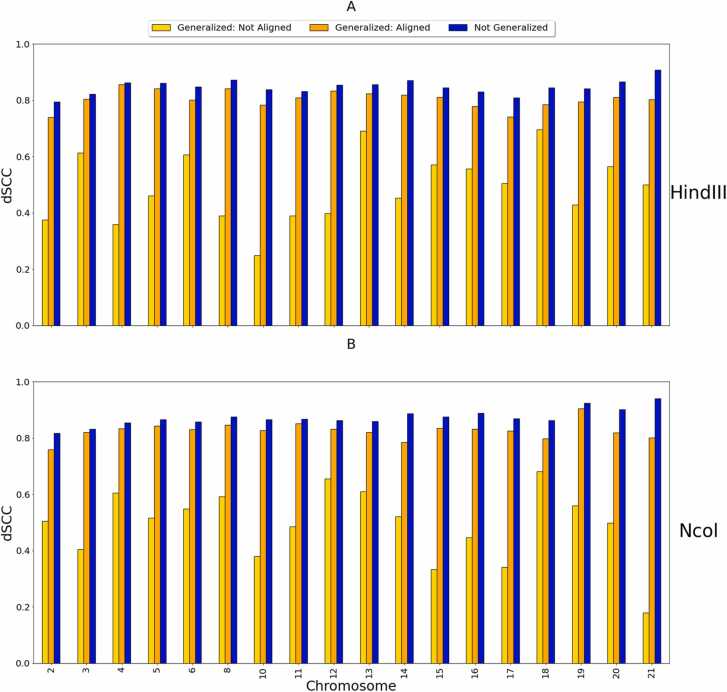
dSCC comparison: generalized and non-generalized models for *Hin*dIII (A) and Ncol (B) restriction enzymes. The figure shows the dSCC values for generalized and non-generalized HiC-GNN models for the *Hin*dII (A) and Ncol (B) restriction enzymes both with and without aligned node embeddings.

**Fig. 15:**
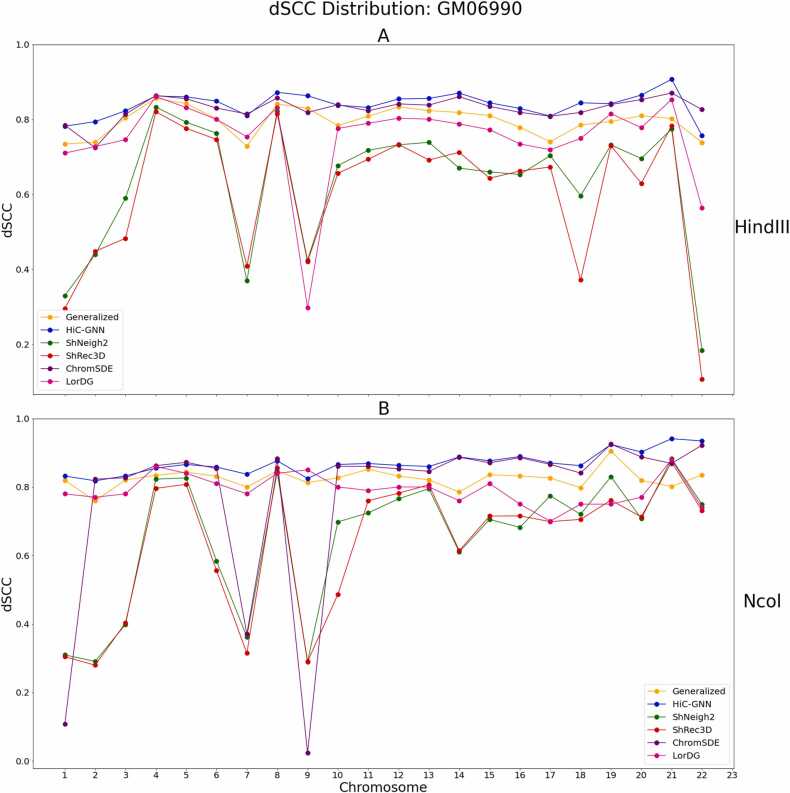
dSCC comparison: *Hin*dIII (A) and Ncol (B) restriction enzymes. The figure shows a comparison of HiC-GNN with the other methods on the *Hin*dII (A) and Ncol (B) GM06990 data.

To further test our method, we validated on the GM06990 cell line as well. This data consists of 22 Hi-C maps generated from the *Hin*dIII and Mbol restriction enzymes at 1 mb resolution. We also tested how well HiC-GNN can generalize across input restriction enzymes. The choice of restriction enzyme in the Hi-C experiment leads to variability in the resulting Hi-C data [48], [49], [50]. Thus, accurate generalizability across restriction enzymes would show that our method is robust to this variation. We tested this generalization by training a model on one restriction enzyme and testing on another, following the same alignment protocol as in the test for generalization across input resolution. The results of these tests along with comparisons with the other methods can be found in Fig. 14, Fig. 15.

From these figures, it is clear that the non-generalized HiC-GNN is either on par with or outperforms the other four methods. Moreover, the generalized HiC-GNN models outperform ShNeigh2, ShRec3D, and LorDG on most of the chromosomes despite being trained on data generated using an entirely different restriction enzyme. Since our method accurately generalizes across restriction enzymes, the models learned by HiC-GNN are robust to the variation in data caused by different choices of restriction enzymes in the Hi-C experiment. This generalization is likely possible because the distributions of contacts between maps corresponding to different restriction enzymes are similar enough for the neural network to generalize despite the variation in the input data.

**Fig. 16:**
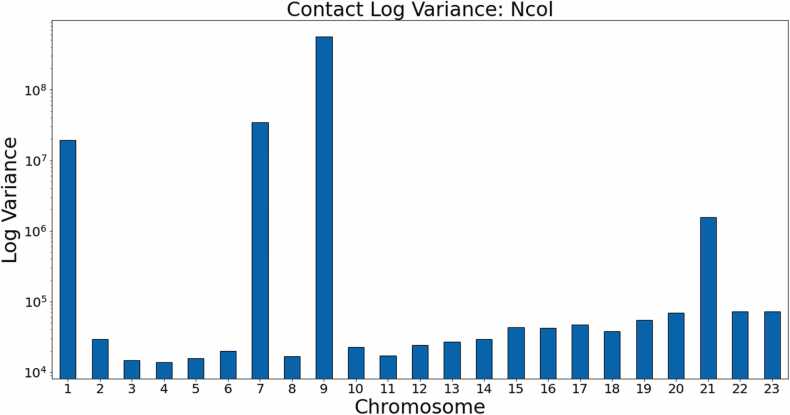
Contact variances: GM06990 Ncol data. The figure shows the log-variances of the contacts for each chromosome. Chromosomes with higher contact variances lead to lower dSCC values for the other methods, whereas HiC-GNN is relatively robust to high contact variance. This is particularly notable on chromosomes 1, 7, 9, and 21.

**Fig. 17:**
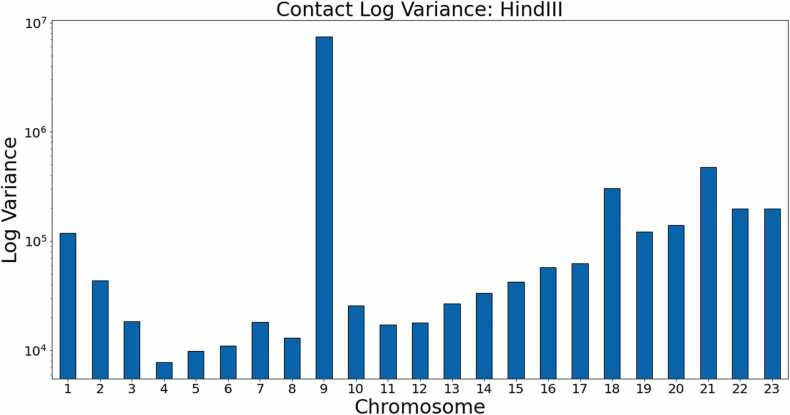
Contact variances: GM06990 *Hin*dIII data. The figure shows the log-variances of the contacts for each chromosome. Chromosomes with higher contact variances lead to lower dSCC values for the other methods, whereas HiC-GNN is relatively robust to high contact variance. This is particularly notable on chromosomes 1, 9 and 18.

**Fig. 18:**
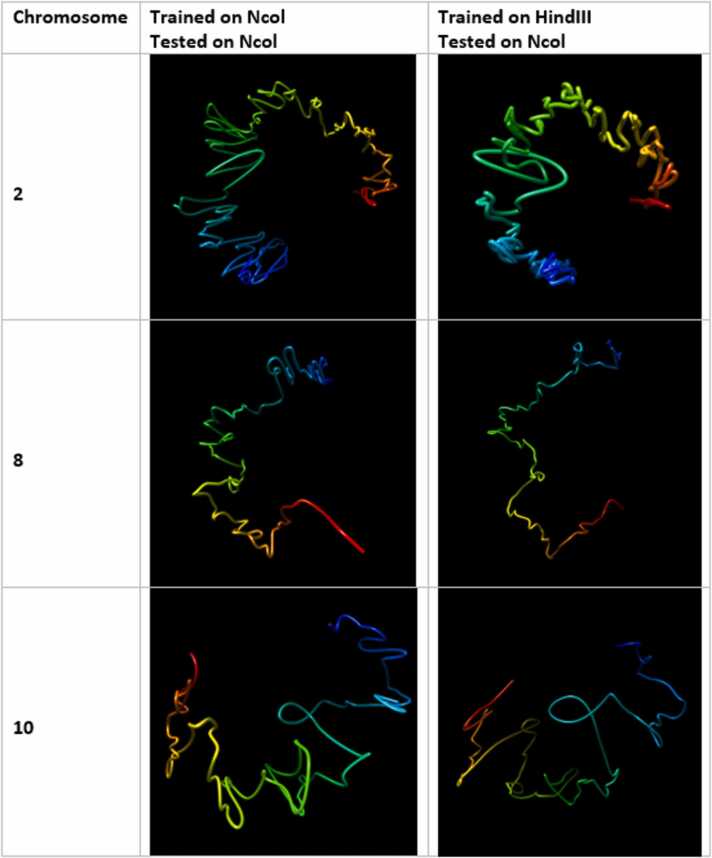
Visual comparison of structures generated from HiC-GNN generalized across restriction enzymes. The first column lists the chromosomes for which the 3D structure prediction was done. The second column shows structures generated from a model trained and tested on the Ncol maps. The third column shows structures generated from a model trained on the *Hin*dIII maps and tested on the Ncol maps.

**Fig. 19:**
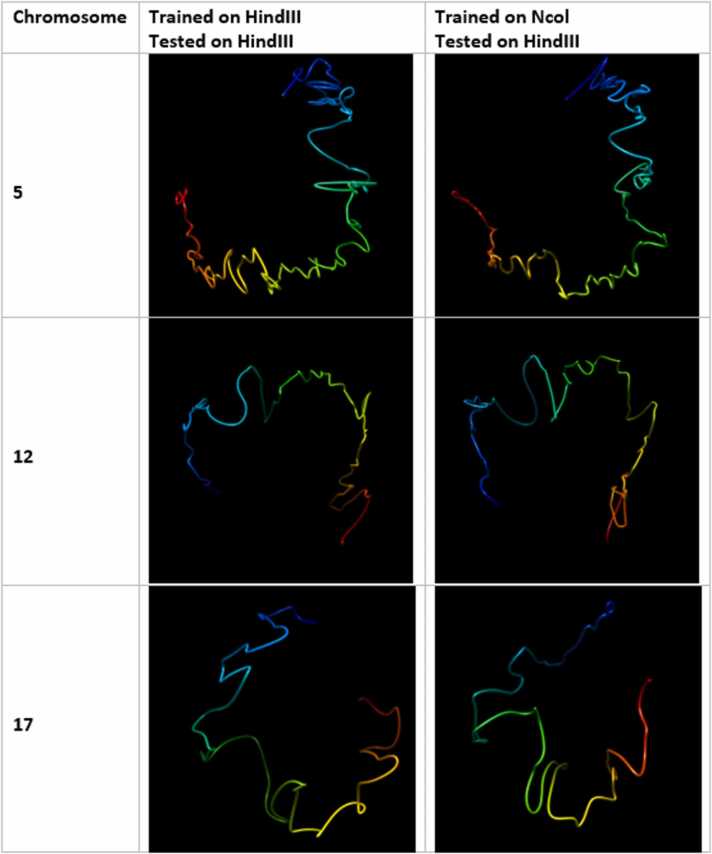
Visual comparison of structures generated from HiC-GNN generalized across restriction enzymes. The first column lists the chromosomes for which the 3D structure prediction was done. The second column shows structures generated from a model trained and tested on the *Hin*dIII maps. The third column shows structures generated from a model trained on the Ncol maps and tested on the *Hin*dIII maps.

Fig. 16, Fig. 17 show the log variance for the contacts within these data sets. Once again, the performance of the other methods is severely affected by high variance in the input contact maps, particularly in chromosomes 9, 7, and 1 in the Ncol data and chromosomes 18, 9, and 1 in the *Hin*dIII data. Fig. 18, Fig. 19 provide a visual comparison between structures generated from a generalized HiC-GNN model and a non-generalized HiC-GNN model for three randomly selected chromosomes.

### 4.7 K562 cell line dataset

#### 4.7.1 Generalization 3: generalization across cell populations

Finally, we tested how well HiC-GNN could generalize across different cell populations, i.e., how well can HiC-GNN perform when trained and tested across the Hi-C maps of chromosomes generated from separate Hi-C experiments. For this test, we utilized the K562 cell line from [32]. This data set consists of several sets of Hi-C maps generated from separate Hi-C experiments, each of which containing 23 chromosomes at 1 mb resolution. We will refer to each of these orthogonal sets as replicates. Each replicate was generated using a separate population of cells. Due to variability in the size of the cell populations, each replicate has a varying quantity of total contacts across the entire genome. The smallest number of total contacts in the replicate sets is 53 million, and the largest number is 310 million. In most applications of Hi-C data analysis, it is typical to analyze the combination of all replicate maps corresponding to multiple Hi-C experiments.

In this test, we consider maps of two levels of total contacts: full coverage and half coverage. The full coverage maps are derived by taking the element-wise sum of each replicate map within the data set for each distinct chromosome. The half coverage maps are derived by taking the same element-wise sum except using only three of the six replicate maps within the data set. The three maps used for the half coverage maps along with the total contacts (across all chromosomes) for each map are shown in Table 7.

**Table 7:** Comparison of total contact frequencies across the entire genome for the half and full coverage maps.

Map Name	Number of Contacts (Millions)
HiC072	53
HiC074	65
HiC069	310
Half Coverage	428
Full Coverage	932

The half coverage map corresponds to the element-wise sums of HiC02, HiC074, and HiC069. The full coverage map corresponds to the element-wise sums of each map within the dataset.

**Fig. 20:**
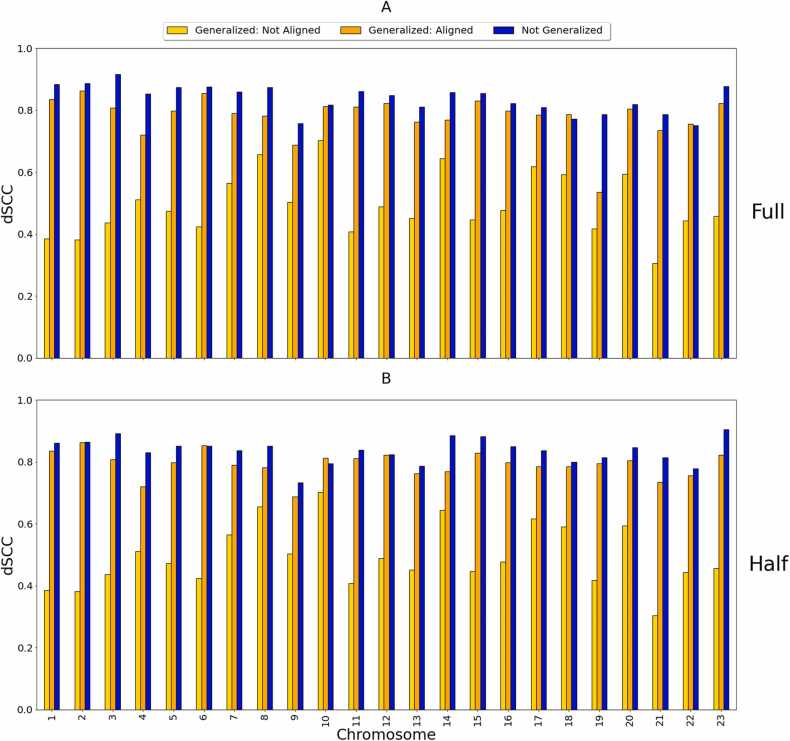
dSCC comparison: generalized and non-generalized models for full (A) and half (B) coverage. The figure shows the dSCC values for generalized and non-generalized HiC-GNN models at full (A) and half (B) coverage both with and without aligned node embeddings.

**Fig. 21:**
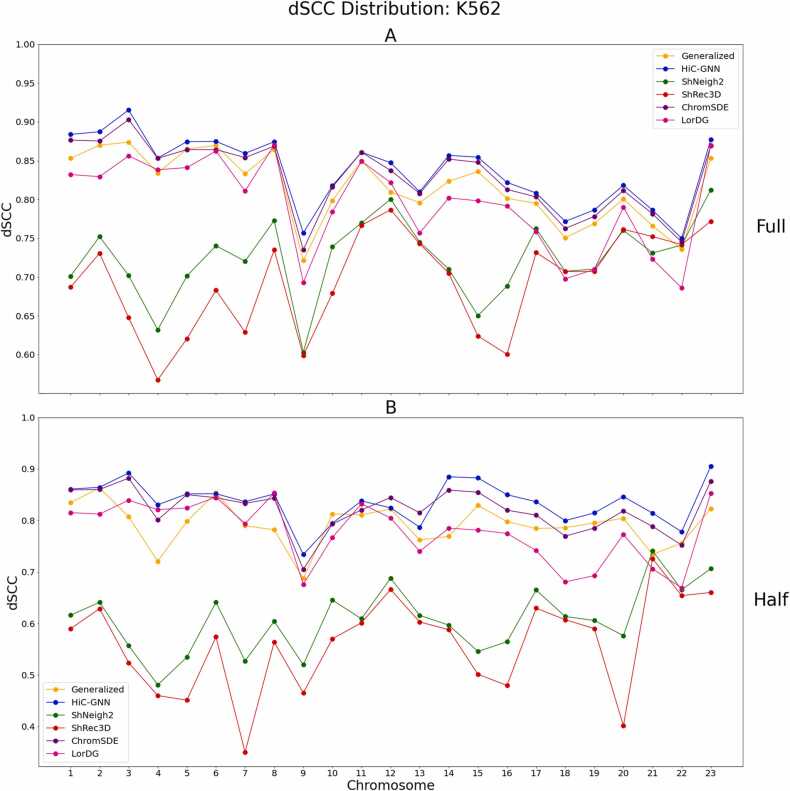
dSCC comparison: half to full (A) and full to half (B) coverage. The Fig. A shows the results of training HiC-GNN on maps with half coverage and testing on the full coverage map. The Fig. B shows the results of training HiC-GNN on maps with full coverage and testing on the half coverage map.

**Fig. 22:**
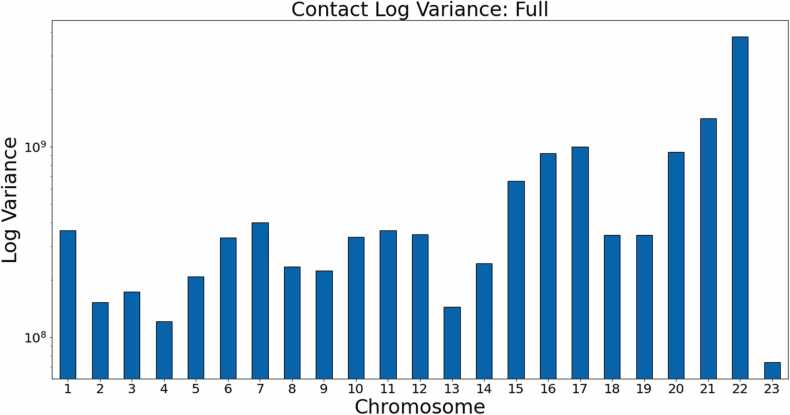
Contact variances: K562 full coverage. The figure shows the log-variances of the contacts for each chromosome. Chromosomes with higher contact variances lead to lower dSCC values for the other methods, whereas HiC-GNN is relatively robust to high contact variance.

**Fig. 23:**
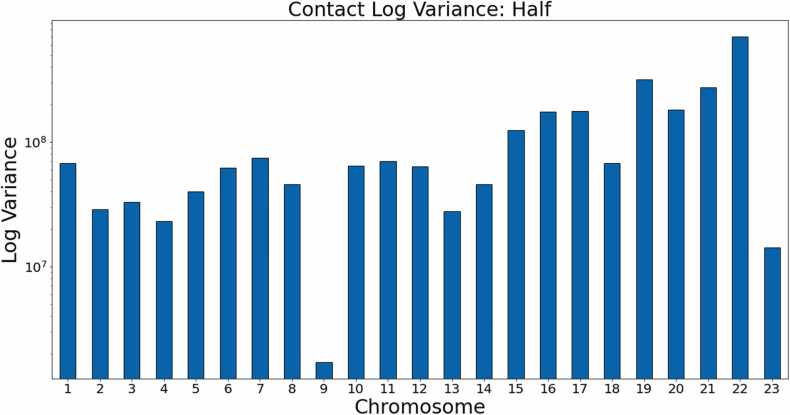
Contact variances: K562 half coverage. The figure shows the log-variances of the contacts for each chromosome. Chromosomes with higher contact variances lead to lower dSCC values for the other methods, whereas HiC-GNN is relatively robust to high contact variance.

In this experiment, we trained HiC-GNN on the half coverage maps and generalized the resulting model to the full coverage maps to test how well HiC-GNN generalizes from contact-sparse data–which we called the Half to Full Coverage generalization. We also trained HiC-GNN on the full coverage maps and generalized the resulting model to the half coverage maps to test how well HiC-GNN generalizes to contact-sparse data– which we called the Full to Half Coverage generalization). The results of these comparisons are seen in Fig. 20, Fig. 21 The contact variances for the full and half coverage maps can be found in Fig. 22, Fig. 23 respectively.

**Fig. 24:**
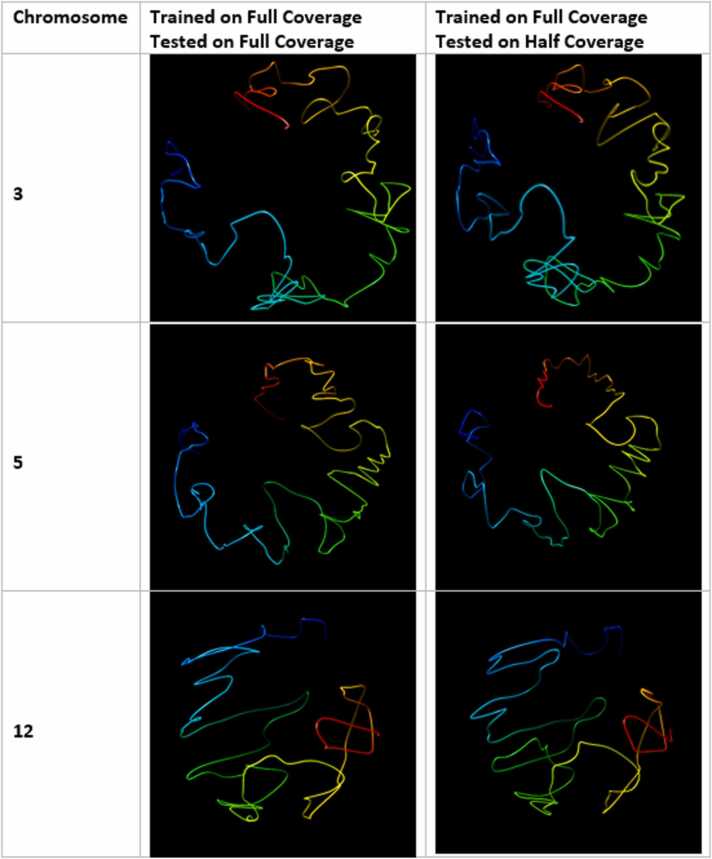
Visual comparison of structures generated from HiC-GNN generalized across cell populations. The first column lists the chromosomes for which the 3D structure prediction was done, the second column shows the structures generated from a model trained and tested on a full coverage map, and the third column shows structures generated from a model trained on a full coverage map and tested on a half coverage map.

From these figures, one can tell that HiC-GNN can indeed generalize from maps containing higher degrees of contact sparsity. Although there is some drop in the reconstructive performance of HiC-GNN associated with generalizing on data containing fewer contacts, it is important to note that the generalized models were either trained or tested on data containing less than half of the contacts as the non-generalized models. Fig. 24 gives a visual comparison of the structures generated with generalized HiC-GNN models and structures generated using non-generalized HiC-GNN models.

## 5 Conclusions

In this paper, we presented a novel technique for predicting the 3D structure of chromosomes from Hi-C data using a node embedding algorithm and graph convolutional neural networks. Unlike other typical methods for chromosome structural inference, our method has the capability of generalizing across resolutions, restriction enzymes, and cell populations. We also showed that the performance of our method is superior when compared with other methods across multiple data sets. To our knowledge, the generalizations provided by our method are not present in any current methods for chromosome structure prediction.

Our method can generalize for three reasons. Firstly, since we generate static node features corresponding to each locus prior to training, we can store the trained parameters of the neural network to be used for inference on unseen data. To our knowledge, all other methods for chromosome structure prediction from Hi-C data treat the coordinates of each locus as the trainable parameters, thereby making it impossible to utilize the trained parameters for inference on new data. Secondly, the node features that we create are similar enough across datasets for the outputs of the neural network to be consistent. Specifically, the increase in reconstructive accuracy following this embedding alignment procedure suggests that the node embeddings corresponding Hi-C maps are approximately isometric. This isometry allows the pre-trained parameters of the neural network to be well-adapted to the distribution of the loci representations of unseen data. Finally, although there exists variation between the training and testing Hi-C maps in general, the distribution of contacts is similar enough for this variation to be smoothed by the neural network. When generalizing across resolution, the variation is caused by difference in the granularity of observed contacts. When generalizing across restriction enzymes, the variation is caused by biases from the Hi-C experiment. When generalizing across cell populations, this variation is caused by sparsity of the data. Although these variations are present, the neural network is still able to generalize. This generalizability is one of the great successes of deep learning, which is why its use in our algorithm is particularly valuable.

Beyond generalizability, there are also several possible advantages to using GCNNs for the task of chromosome structure prediction that were not explored in this paper. For example, batching procedures could be used to improve the training process and more sophisticated embedding alignment could improve the reconstructive performance of generalized models. The batching and parallelization capabilities of graph neural networks could potentially be useful for structural prediction on very high (<10 kb) resolution data. Moreover, the generalizability of our method could also reduce the computational requirements of generating structures on high resolution data via pre-training on low resolution data. We consider these rich directions of our method for future work.

## Funding

This work was supported by the 10.13039/100000001National Science Foundation (NSF) CRII award (grant no: 2153205) to O.O. and start-up funding from the 10.13039/100010174University of Colorado, Colorado Springs to O.O.

## CRediT authorship contribution statement

**Van Hovenga**: Conceptualization, Methodology, Software, Investigation, Data curation, Analysis, Writing – original draft, Writing – review & editing, Validation. **Jugal Kalita**: Methodology, Writing – review & editing. **Oluwatosin Oluwadare**: Conceptualization, Data curation, Investigation, Analysis, Writing – review & editing, Supervision, Resources, Project administration, Funding acquisition.

## Declaration of Competing Interest

The authors declare that they have no known competing financial interests or personal relationships that could have appeared to influence the work reported in this paper.

## Data Availability

All our source codes and data are available at https://github.com/OluwadareLab/HiC-GNN, and is made available as a containerized application that can be run on any platform. We utilized a TensorFlow implementation of LINE available at https://github.com/shenweichen/GraphEmbedding.
